# Dataset on quality parameters of fertilized eggs stored for one week at 12 °C and 80% relative humidity, with or without 2% CO₂, and after a 24-hour acclimatization at 20 °C

**DOI:** 10.1016/j.dib.2026.112600

**Published:** 2026-02-16

**Authors:** Pauline Javaloyes, Anne Collin, Sophie Réhault-Godbert

**Affiliations:** INRAE, Université de Tours, BOA, 37380 Nouzilly, France

**Keywords:** Chicken, Storage, Albumen, pH, Haugh units, Yolk index, Eggshell integrity, Dehydration

## Abstract

In hatcheries, hatching eggs are commonly stored before incubation in order to facilitate the synchronization of hatchings and to meet customer demand for chicks. Recommendations for long storage (>15 days) is to store egg at temperature ≤12 °C, 80 % relative humidity to maintain internal egg quality without affecting embryo viability. In this data paper, we provide data related to the evolution of quality parameters of hatching eggs from the day of lay up to seven days of storage at 12 °C, 80 % relative humidity, with or without 2 % CO₂. Several egg quality parameters including albumen pH, Haugh units, yolk index and color, eggshell breaking strength, and percentage of eggshell. The distribution of eggs by batch, the description of quality parameters and their raw values are available as excel files (Research Data Gouv repository). The quality parameters of eggs (*n* = 15 per condition) were measured on the day of lay, and after one, three, five, six, and seven days of storage. The article also includes a comparison of the quality of eggs stored for six days with or without a 24-hour thermal acclimatization. Statistical analysis (Kruskal-Wallis followed by Dunn tests for multiple comparisons, *p* < 0.05) showed that the overall quality of albumen was higher for eggs stored under CO_2_ conditions compared to control eggs (higher Haugh units, lower pH). These values were not significantly different from those from eggs analyzed on the day of lay. While the pH of eggs stored at 12 °C and 80 % relative humidity (control conditions) gradually increased over storage, the pH of eggs stored in the same thermal conditions than controls but with 2 % CO_2_ remained stable throughout the storage period. Haugh units were significantly lower in eggs stored under control conditions from day three of storage onwards compared with 2 % CO_2_ eggs and unstored eggs (freshly laid eggs). Regardless of the condition, eggs exhibited a loss in egg weight during storage with no significant difference observed between conditions. None of the other parameters (yolk index and color, eggshell strength and thickness) showed any statistically significant differences between groups. Furthermore, the statistical analysis showed that a 24-hour thermal acclimatization induced a higher loss of egg weight than in eggs stored for six and seven days under control or under 2 % CO_2_. None of the other parameters was affected when comparing values of acclimated eggs to those of the eggs stored under control conditions or under 2 % CO_2_ for six or seven days.

These data can be reused as a reference to optimize the storage conditions of fertilized eggs from chicken layer breeds, in order to maintain egg quality during storage and improve the viability of chicken embryos and their hatchability. It also highlights the effect of thermal acclimatization for 24 h on the internal quality of eggs.

**Table:** Specifications Table

Subject	Biology
Specific subject area	Short-term egg storage, 2 % CO_2_ enrichment and acclimatization on the quality of fertilized egg
Type of data	Workflow, Table, Graph, Figure Analyzed, Processed
Data collection	Two hundred eggs were candled, weighed individually (Sartorius Practum balance, Sartorius, Gottingen, Germany), and distributed in 13 distinct batches of similar weight means (T-test). Eggs were stored in climatic chambers (Memmert ICH 260, Schwabach, Germany) at 12 °C, 80 % relative humidity, 50 % ventilation and ± 2 % CO_2_), in egg plastic trays, every two cells to allow for homogenous atmosphere around each egg. Acclimatization of eggs prior to incubation was performed for 24 h at 20 °C in an air-conditioned room. The egg weight loss was obtained by weighing eggs before and after storage. Eggshell strength, yolk color and index, and Haugh units of eggs were measured using a digital egg tester (DET6000, Nabel, Kyoto, Japan). The pH of the egg white was measured with a pH-meter (GLP Sension+ Ph31, Hach, Lognes, France). Eggshell weight was measured after drying for two hours at 110 °C in an oven (AC120, FIRLABO SA, Mézieux, France).
Data source location	INRAE, Université de Tours, BOA, Nouzilly, France
Data accessibility	Repository name: The results of the script used to distribute eggs in batches and raw data of the experiment are available as open data in the Research Data Gouv repository https://doi.org/10.57745/D1FAHU.
Related research article	None

## 1 Value of the Data

• Finding conditions to store fertilized eggs without compromising embryo viability during incubation is of major importance for the sustainability of chick production
• Most research on egg storage conditions involving CO_2_ and low temperature is based on old publications that used laying breeds that are very different from those resulting from genetic selection on egg quality and production traits
• The experiment aimed to explore the effect of 2 % CO_2_ at low storage temperature during the first week of storage on the quality of egg laid by a commercial laying breed
• The experiment was designed to evaluate in parallel the effect of 24-hour acclimatization at 20 °C on egg quality
• The effect of storage on egg quality has been assessed via multiple parameters and data were analyzed using principal component analysis and statistical analyses to compare conditions
• The protocol was designed to limit any experimental bias and ensure the reliability of the observations

## 2 Background

Storing eggs before incubation is a common practice that facilitates logistics in hatcheries [1]. However, storage for >14 days significantly impairs egg quality due to the diffusion of gases (water and CO_2_) through the eggshell [2]. Water loss reduces egg weight while decreasing the amount of water available for the embryo development. The diffusion of the carbon dioxide naturally present in the egg white causes the increase in the pH of the albumen and its liquefaction (decrease in Haugh units) [3]. This process impairs the vitelline membrane (decrease in yolk index) [4] and blastoderm cells [5,6], and therefore the development and viability of the embryo. For long-term storage, it is recommended to store eggs at 12 °C [3,6,7]. The disadvantage of this low-temperature storage is the need to perform warming of eggs prior to incubation to avoid thermal shock. A complementary strategy is to prevent the diffusion of CO_2_ by enriching the storage atmosphere with CO_2_ [8,9]. We used a combination of temperature (12 °C) and CO_2_ (2 %) to explore to what extend the quality of freshly laid eggs from a commercial layer breed is maintained during a short period of storage, and if some quality parameters are changing after a 24-h acclimatization.

## 3 Data Description

After candling and weighing, the eggs were divided into 13 batches of 15 eggs so that all batches had a similar mean weight (T-test, no statistical difference between the mean weights of the batches [10]). The distribution of eggs into batches, the p-values between batches, and all data collected during the experiment are available in the INRAE repository [11]. The distribution of eggs into batches is indicated in the “Experimental design, materials and methods” section. Some eggs had to be replaced with additional eggs of similar weight due to human handling errors or shell breakage that had not been detected during candling but which were identified due to their very low shell strength values. However, the T-test performed on the modified batches showed no significant difference between the average weights of the batches. This verification was important to limit any bias in the data that could result from differences in mean weight between batches and not from experimental conditions.

**Fig. 1:**
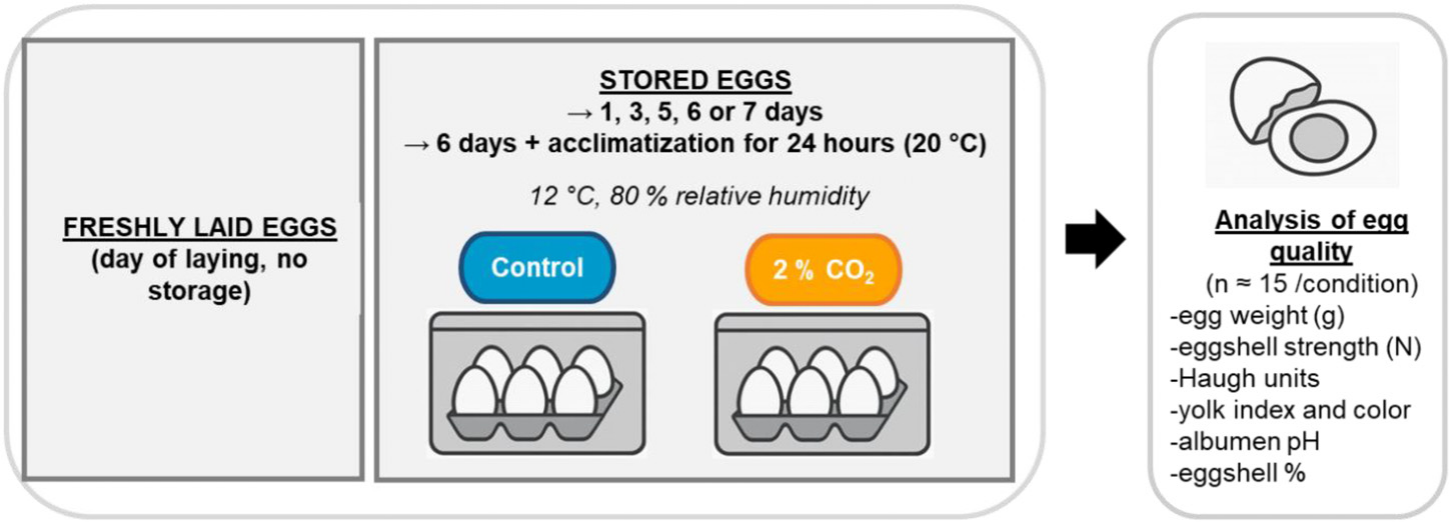
General outline of the experimental protocol. Freshly laid eggs were all weighted, distributed into 13 various batches of 15 eggs (*n* = 15). One batch of eggs was dedicated to quality analysis the day of lay, and the other 14 batches were stored before their quality analysis. For storage, eggs were randomly distributed in ventilated climatic chambers (12 °C, 80 % relative humidity) under either control conditions or experimental conditions (2 % CO₂). Control and 2 % CO_2_ eggs were stored for one, three, five, six or seven days before analysis. In parallel, some eggs were stored in control or 2 % CO_2_ climatic chambers for six days and then acclimated for 24 h at 20 °C before analysis.

Fig. 1 presents the workflow of the experimental protocol. It includes a quality assessment performed on freshly laid eggs and during one week of storage either under control conditions or under 2 % CO₂ enrichment. In addition, we compared the quality of eggs after 6 or 7 days of storage in climatic chambers to eggs acclimated for 24 h at 20 °C.

The data description is structured in two parts: the effect of storage duration and conditions, and the effect of acclimatization, on egg quality.

**Fig. 2:**
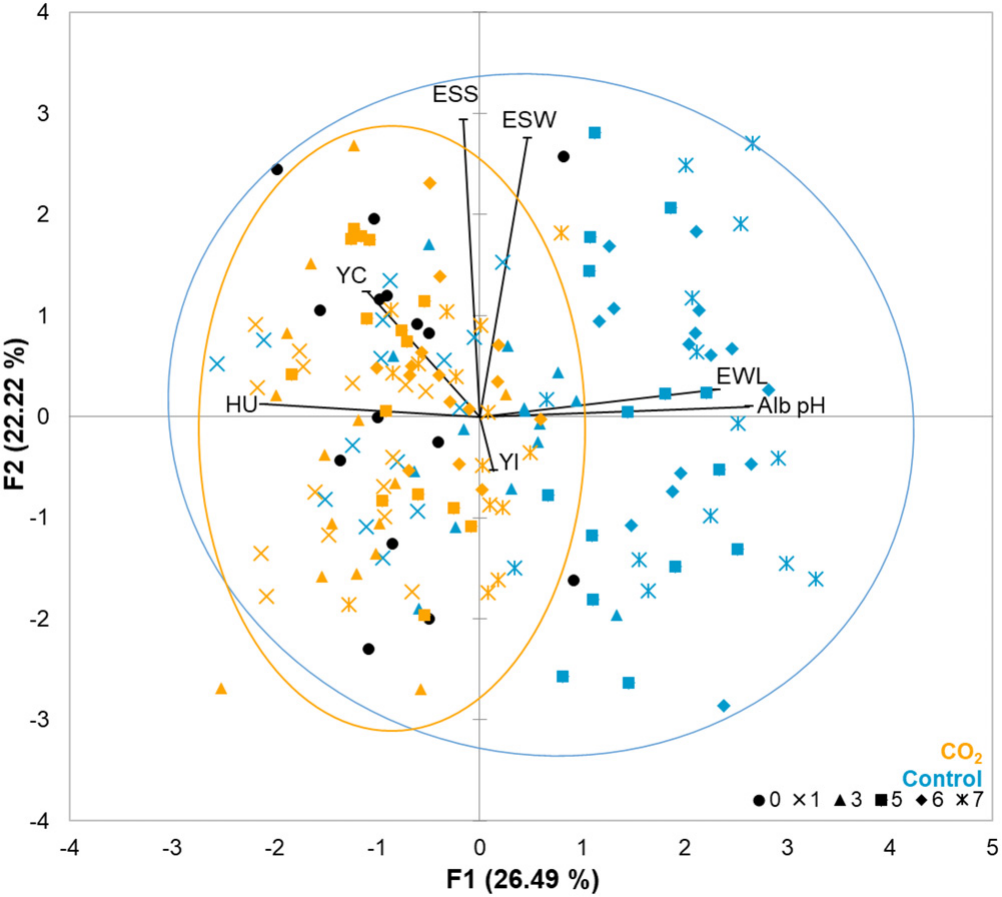
Principal Component Analysis (PCA) corresponding to egg quality parameters of unstored egg and eggs stored for seven days of storage, under control or 2 % CO_2_ conditions at 12 °C and 80 % relative humidity. The first two principal components (F1 and F2) explained 26.5 % and 22.2 % of the total variance, respectively. Vectors represent the contribution and correlation of each parameter to the PCA dimensions. Eggs are colored according to experimental conditions: control group (orange) and 2 % CO₂ group (blue). Eggs are also represented by different symbols according to their storage duration: unstored eggs (circle, 0), eggs stored for one day (cross), three days (triangle), five days (square), six days (diamond), or seven days (star). The quality parameters included Haugh units (HU), yolk index (YI), yolk color (YC), albumen pH (Alb pH), egg weight loss (EWL), eggshell strength (ESS), and eggshell weight (ESW). Orange and blue ellipsoids of control and CO_2_ conditions are defined by a 95 % confidence interval.

Fig. 2 presents the Principal Component Analysis (PCA) performed on all eggs stored or not under the two experimental conditions and across all storage durations. The variables that contributed to F1 axis were related to albumen quality (Haugh units or HU; pH of albumen or Alb pH) and egg weight loss (or EWL), while those contributing to the F2 axis were mainly related to eggshell quality (Eggshell strength or ESS; eggshell weight or ESW). The results indicated that control and CO_2_ eggs were grouped depending on the condition (control or CO_2_), samples from the control group being essentially gathered on the right side of the graph and those from the 2 % CO_2_ group being clustered on the left. However, the distribution of control eggs was more dispersed than 2 % CO_2_ eggs, as illustrated by the ellipsoids, with eggs stored for one or three days in the control conditions appearing on the left together with 2 % CO_2_ eggs. Overall, the results indicated that 2 % CO₂-stored and fresh eggs (unstored, black circles) tend to exhibit higher Haugh units and lower albumen pH, whereas control eggs show the opposite trend.

Results were further analyzed using the Kruskal-Wallis test (Table 1), which, when significant, was followed by a Dunn’s post-hoc test for multiple comparisons.

**Table 1:** Egg quality data of unstored eggs and stored eggs during one week of storage under control or 2 % CO₂ conditions.

Storage (days) and condition		Albumen pH	Haugh units	Yolk index	Yolk color	Eggshell breaking strength (N)	Eggshell weight (%)	Egg weight loss (%)
0	N/A	8.10 ± 0.18	96.9 ± 3.9	0.48 ± 0.02	5.48 ± 0.35	48.0 ± 4.8	10.2 ± 0.8	N/A
1	**Control**	8.39 ± 0.22	94.1 ± 4.1	0.47 ± 0.02	5.61 ± 0.30	47.3 ± 4.8	10.2 ± 0.5	0.07 ± 0.01
	**2 % CO_2_**	8.01 ± 0.14	92.3 ± 3.6	0.48 ± 0.03	5.65 ± 0.40	44.6 ± 5.4	10.0 ± 0.6	0.07 ± 0.01
3	**Control**	8.76 ± 0.16	93.6 ± 4.9	0.49 ± 0.03	5.51 ± 0.26	45.1 ± 4.6	10.2 ± 0.4	0.18 ± 0.02
	**2 % CO_2_**	7.98 ± 0.08	95.0 ± 4.7	0.48 ± 0.02	5.48 ± 0.34	46.0 ± 6.5	10.1 ± 0.6	0.13 ± 0.02
5	**Control**	8.91 ± 0.13	88.1 ± 4.6	0.47 ± 0.02	5.42 ± 0.35	44.9 ± 7.0	10.1 ± 0.8	0.28 ± 0.05
	**2 % CO_2_**	8.07 ± 0.13	93.8 ± 4.6	0.47 ± 0.02	5.79 ± 0.39	47.1 ± 4.3	10.3 ± 0.5	0.21 ± 0.03
6	**Control**	9.06 ± 0.10	88.7 ± 5.0	0.48 ± 0.01	5.45 ± 0.33	47.0 ± 5.9	10.3 ± 0.6	0.38 ± 0.06
	**2 % CO_2_**	8.11 ± 0.07	92.2 ± 3.8	0.46 ± 0.02	5.63 ± 0.30	48.7 ± 4.5	10.2 ± 0.5	0.26 ± 0.03
7	**Control**	9.02 ± 0.11	89.7 ± 4.5	0.47 ± 0.03	5.46 ± 0.37	46.3 ± 6.6	10.2 ± 0.8	0.42 ± 0.07
	**2 % CO_2_**	8.05 ± 0.15	93.4 ± 4.3	0.47 ± 0.03	5.50 ± 0.38	46.7 ± 4.5	10.0 ± 0.7	0.34 ± 0.05
*P-value*		**< 0.0001**	**< 0.0001**	0.0975	0.1720	0.5767	0.9203	**< 0.0001**

For each parameter measured, mean values ± standard deviations are indicated. The statistical analysis was performed using a Kruskal–Wallis test (*p* < 0.05). N/A, non-applicable. When significant, p-values are shown in bold.

**Fig. 3:**
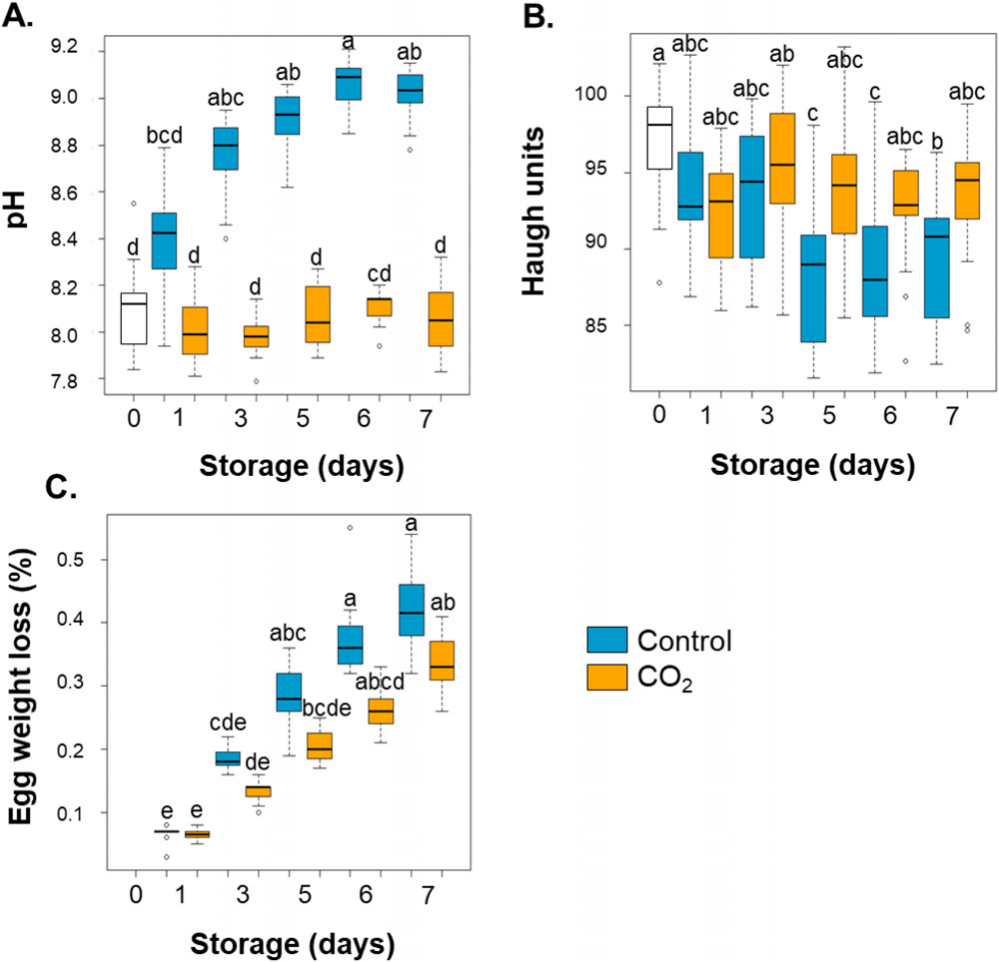
pH, Haugh units and egg weight loss of unstored eggs and eggs during one week of storage under control and 2 % CO₂ conditions. A. Albumen pH. B. Haugh units. C. Egg weight loss (%). Eggs were analyzed on the day of laying (white bars) and after one, three, five, six and seven days of storage at 12 °C and 80 % relative humidity, under control conditions (blue bars) or with 2 % CO₂ (orange bars). Boxplots with different letters were significantly different according to Dunn’s post hoc test (p < 0.05).

As presented in Table 1, the quality parameters showing significant differences were albumen pH, Haugh units, and percentage of egg weight loss, which corresponded to variables contributing to F2 axis of the PCA (Fig. 2). The post-hoc analysis (Fig. 3) showed a significant difference in albumen quality and egg weight loss over time in control conditions compared to 2 % CO₂ conditions (Fig. 3A, B, and C, respectively). Under 2 % CO₂ conditions, the pH of the albumen remained stable and its value was not different from that of freshly laid eggs (Fig. 3A). Under control conditions, pH increased up to three days of storage to reach a plateau (Fig. 3A). Under control conditions, Haugh units were significantly lower after five days of storage compared to the day of lay while under 2 % CO₂ conditions, Haugh units were not different from the value of freshly laid eggs (Fig. 3B). Under both control and 2 % CO_2_ conditions, the egg weight loss increased over time (Fig. 3C).

In the second study, we compared the quality of eggs stored for six days under control and 2 % CO_2_ conditions after a 24-hour thermal acclimatization at 20 °C to that of eggs stored for six and seven days under the same conditions but without acclimatization.

**Fig. 4:**
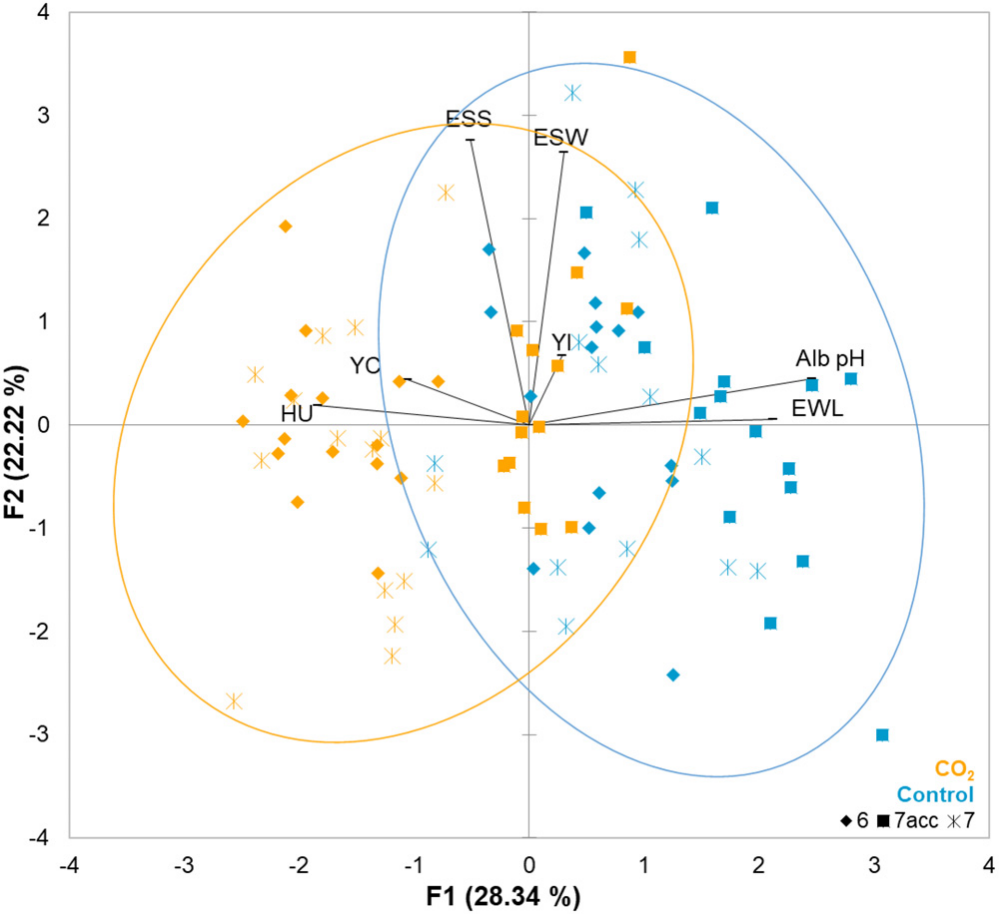
Principal Component Analysis (PCA) corresponding to quality parameters after six and seven days of storage, and six days of storage under control or 2 % CO_2_ conditions followed by 24 h of acclimatization. The first two principal components (F1 and F2) explained 28.3 % and 22.2 % of the total variance, respectively. Vectors represent the contribution of each parameter to the PCA dimensions. Eggs are colored according to experimental conditions: control group (orange) and 2 % CO₂ group (blue). Eggs are also represented by different symbols according to their storage duration: six days (diamond), six days followed by 24 h of acclimatization (square) or seven days (star). The quality parameters included Haugh units (HU), yolk index (YI), yolk color (YC), albumen pH (Alb pH), egg weight loss (EWL), eggshell strength (ESS), and eggshell weight (ESW). Orange and blue ellipsoids, of control and CO_2_ conditions are defined by a 95 % confidence interval.

In Fig. 4, the second PCA representation, the variables contributing to the F1 and F2 axes were the same as in the first PCA (Fig. 2). Acclimated eggs (orange squares) after six days under 2 % CO_2_ environment were centered with eggs stored for six days without 2 % CO_2_ (control, blue diamonds), while eggs stored for six or seven days in this condition were clustered on the left. In contrast, acclimated eggs from the control conditions were essentially located on the right.

As in Study 1, egg quality parameters were analyzed using non-parametric statistical tests. Comparison between groups were performed using the Kruskal-Wallis test (Table 2), which, when significant, was followed by a Dunn’s post-hoc test for multiple comparisons.

**Table 2:** Egg quality data after six and seven days of storage under control or 2 % CO₂ conditions and after a six-day storage in both conditions followed by a 24-hour acclimatization at 20 °C.

Storage (days) and condition		Albumen pH	Haugh units	Yolk index	Yolk color	Eggshell breaking strength (N)	Eggshell weight (%)	Egg weight loss (%)
**6**	**Control**	9.06 ± 0.10	88.7 ± 5.0	0.48 ± 0.01	5.45 ± 0.33	47.0 ± 5.9	10.3 ± 0.6	0.38 ± 0.06
	**2 % CO_2_**	8.11 ± 0.07	92.2 ± 3.8	0.46 ± 0.02	5.63 ± 0.30	48.7 ± 4.5	10.2 ± 0.5	0.26 ± 0.03
**7acc**	**Control**	9.15 ± 0.07	86.7 ± 4.8	0.46 ± 0.03	5.25 ± 0.34	46.5 ± 6.2	10.2 ± 0.5	0.61 ± 0.06
	**2 % CO_2_**	8.73 ± 0.14	92.1 ± 3.2	0.47 ± 0.02	5.66 ± 0.32	48.0 ± 5.0	10.2 ± 0.5	0.51 ± 0.05
**7**	**Control**	9.02 ± 0.11	89.7 ± 4.5	0.47 ± 0.03	5.46 ± 0.37	46.3 ± 6.6	10.2 ± 0.8	0.42 ± 0.07
	**2 % CO_2_**	8.05 ± 0.15	93.4 ± 4.3	0.47 ± 0.03	5.50 ± 0.38	46.7 ± 4.5	10.0 ± 0.7	0.34 ± 0.05
*P-value*		**< 0.0001**	**0.0006**	**0.0418**	**0.0162**	0.8287	0.6843	**< 0.0001**

For each parameter measured, mean values ± standard deviations are indicated. Statistical analysis was performed using Kruskal–Wallis test (*p* < 0.05). 7acc, eggs stored for six days then acclimated for 24 h at 20 °C. When significant, p-values are shown in bold.

**Fig. 5:**
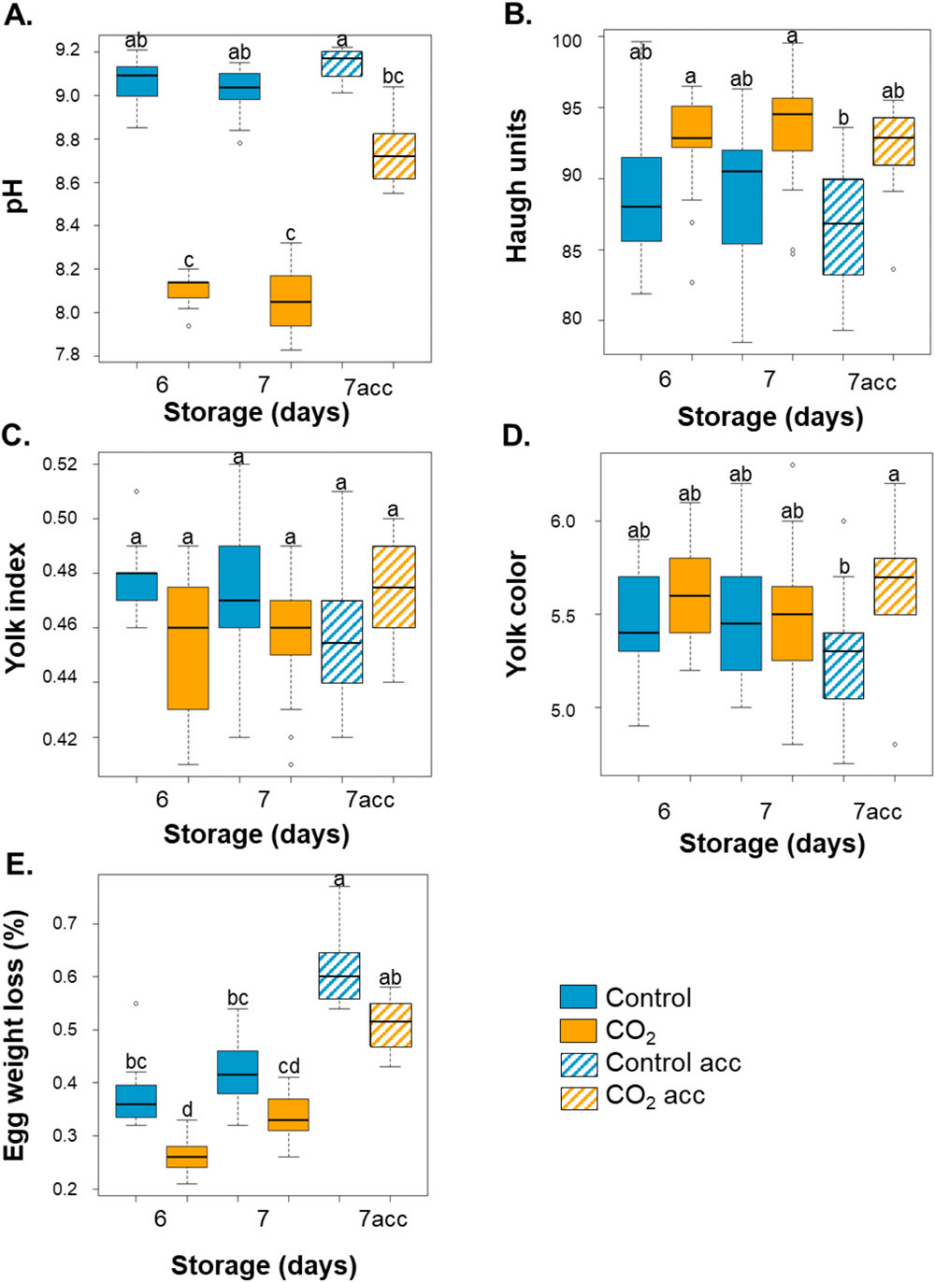
pH, Haugh units, yolk index and color, and egg weight loss after six or seven days of storage under control and 2 % CO₂ conditions followed or not by a 24-hour acclimatization at 20 °C. A. Albumen pH. B. Haugh units. C. Yolk index. D. Yolk color. E. Egg weight loss (%). Eggs were analyzed after six or seven days of storage at 12 °C and 80 % relative humidity, or after six days followed by 24 h of acclimatization (7acc, striped bars) at 20 °C. Eggs under control conditions are illustrated in blue and eggs under 2 % CO₂ are in orange. Boxplots with different letters are significantly different according to Dunn’s post hoc test (p < 0.05).

The parameters showing significant differences were pH, Haugh units, yolk index and color (F1 axis), and percentage of egg weight loss (F2 axis) (Fig. 4). The post-hoc analysis (Fig. 5) showed a significant difference in albumen pH and Haugh units and in egg weight loss over time in control conditions compared to 2 % CO₂ conditions (Fig. 3A, B, and C, respectively).

Under control conditions, post-hoc analysis revealed that acclimatization had no significant effect on albumen pH, Haugh units, yolk color and index (Fig. 5A, B, C and D, respectively) but acclimated eggs exhibited an increase in egg weight loss (Fig. 5E). Under 2 % CO_2_, the pH of albumen of acclimated eggs increased but was not significantly different from that of eggs stored for six or seven days without acclimatization (Fig. 5A). This value was not different from control eggs stored for six or seven days (Fig. 5A). For 2 % CO_2_ eggs, no effect of acclimatization was observed for Haugh units (Fig. 5B), yolk index and color (Fig. 5C, D) compared to eggs stored under 2 % CO_2_ for six or seven days without acclimatization. However, egg weight loss was significantly higher for acclimated 2 % CO_2_ eggs versus 2 % CO_2_ eggs stored for six or seven days without acclimatization (Fig. 5E). To sum up, except for egg weight loss, the other parameters of quality of acclimated eggs were not significantly different from those of egg stored for six or seven days, under control and 2 % CO_2_ conditions. In addition, Haugh units, yolk index, egg weight loss of 2% CO_2_ acclimated eggs were not significantly different (Fig. 5B, C, and E) compared with control acclimated eggs, whereas the albumen pH was significantly higher and yolk color significantly lower in control acclimated eggs compared with 2% CO_2_ acclimated eggs (Fig. 5A and D).

## 4 Experimental Design, Materials and Methods

### 4.1 Egg handling

**Fig. 6:**
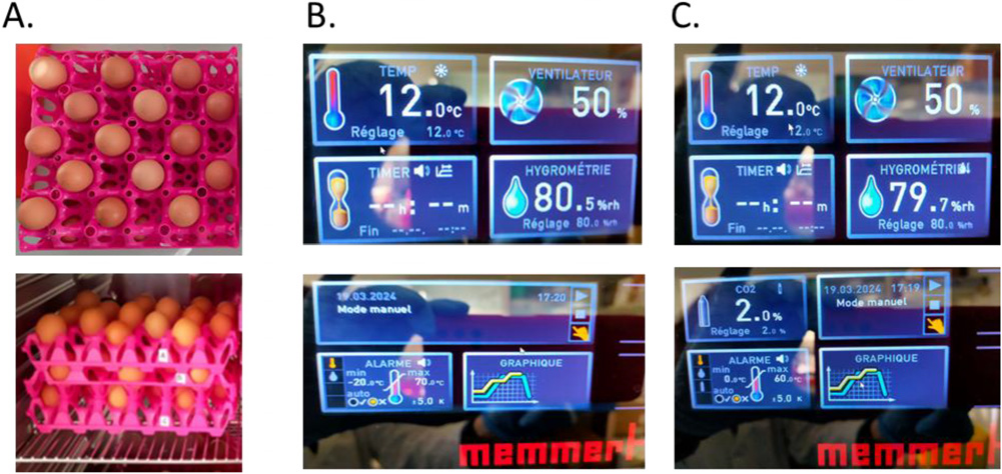
Pictures of the experimental setup in plastic trays and climatic chambers settings screens. A. Distribution of eggs in plastic trays in climatic chambers. Within a climatic chamber, eggs were placed in ascending numerical order, resulting in a random distribution of eggs, independently of their batch number. B. Conditions used for the control chamber. Upper panel: 12.0 °C, ventilation at 50 %, targeted relative humidity: 80 %. Lower panel: no additional CO_2_. C. Conditions used for the CO_2_ chamber. Upper panel: 12.0 °C, ventilation at 50 %, targeted relative humidity: 80 %. Lower panel: 2 % CO_2_. Temperature, relative humidity, and CO_2_ were continuously recorded throughout storage.

Two hundred eggs from the day of lay (Novobrown laying hens, 35 weeks of age) were collected at the hatchery (La Chapelle-sur-Loire, France). Upon arrival at the laboratory, eggs were candled to remove eventual broken eggs and ensure that the air cell was correctly positioned at the top of the egg. Eggs were numbered from 1 to 200 and weighed (Sartorius Practum balance, Sartorius, Gottingen, Germany). To ensure an even distribution of egg weight among the groups, eggs were divided into 13 batches (*n* = 15) of similar weight mean (T-test) [10,11]. Eggs from one batch were immediately analyzed for their quality (unstored eggs, day 0). Six batches were stored in a control climatic chamber (Memmert ICH 260, Büchenbach, Germany) set at 12 °C, 80 % relative humidity, and 50 % ventilation, while six other batches were stored under the same conditions with 2 % CO₂. Five additional eggs were randomly distributed in the climatic chambers with or without 2 % CO_2_ (two under control conditions and three under 2 % CO₂) to eventually replace any eggs that may have been broken and not detected by candling. After each storage period (one, three, five, six and seven days) and for each condition (control and 2 % CO₂), one batch of eggs was analyzed for egg quality. In addition, after six days of storage, one batch from each climatic chamber was placed for 24 h in an air-conditioned room at 20 °C to allow egg warming prior to analysis (to study the effect of thermal acclimatization). All eggs were placed in plastic trays in ascending numerical order, resulting in a random distribution of eggs in the climatic chambers. An empty space was introduced between each egg both horizontally and vertically to allow for proper ventilation around each egg Fig. 6.

### 4.2 Analysis of egg quality

After each storage period, eggs were weighed with the same balance as the day of receipt (Sartorius Practum balance, Gottingen, Germany) and analyzed using the digital egg tester (DET6000, Nabel, Kyoto, Japan). Measurements included egg weight (that is necessary for all other measurements with DET6000 but that was not used to calculate weight loss), Haugh units, yolk color and index. The albumen from each egg was separated from the yolk using an egg separator and transferred to 50 mL Falcon tube to measure the pH (pH meter, GLP Sension+ Ph31, Hach, Lognes, France). The pH value was recorded after stabilization. Eggshells were carefully rinsed with tap water and dried at 110 °C for two hours in an oven (AC120, FIRLABO SA, Mézieux, France), then weighed (Sartorius Practum balance, Gottingen, Germany). To avoid any bias related to handling time, the measurement of egg quality parameters was carried out by alternating eggs from each condition (control, 2 % CO_2_). Egg weight loss was calculated as follows: weight of egg after storage (g)/ weight of egg before storage (g) x 100. The proportion of eggshell to the egg was calculated according to the following equation: weight of dry eggshells (g)/ weight of egg before storage (g) x 100. The description of each parameters and resulting data from all eggs are available in a public data repository [11].

### 4.3 Data analysis

Data were first analyzed using principal component analysis (XLSTAT, Lumivero, Bordeaux, France). Statistical analyses were carried out to evaluate the effect of storage condition, storage duration, and their potential interaction on various egg quality indicators. Outliers were first identified using a dispersion factor of 1.5. Since the data did not meet the criteria for normality and homogeneity of variance, non-parametric tests were applied. Therefore, the interaction between the two factors could not be considered. A Kruskal–Wallis test was performed, followed, in case of significance, by a Dunn’s post-hoc test with Bonferroni correction for multiple comparisons. All analyses were carried out using RStudio software (version 2024.12.0) [12], with the significance threshold set at 5 % (α = 0.05).

## Limitations

We used eggs from a modern breed of laying hens whose females have been selected for decades for egg quality, in order to meet some consumer expectations in terms of eggshell quality and thus limit contaminations by pathogens (*Salmonella enterica* Enteritidis). Eggs from modern laying hens are therefore characterized by increased eggshell strength compared to traditional breeds and those used in the 1960s, particularly due to an increased eggshell weight and thickness [13]. Consequently, the values of the egg quality parameters described in this article after storage or after acclimatization may differ when hens with lower eggshell quality are used (e.g. meat-type breeds, laying hens at the end of their production period [14]).

## Ethics Statement

This work complies with the ARRIVE guidelines [15]. The authors have read and follow the ethical requirements for publication in Data in Brief and confirm that the current work does not involve animal experiments, but only fertilized eggs collected on the day of laying and kept in a state of diapause throughout the storage period.

## CRediT authorship contribution statement

**Pauline Javaloyes:** Methodology, Validation, Formal analysis, Investigation, Writing – original draft, Writing – review & editing, Visualization. **Anne Collin:** Formal analysis, Writing – review & editing. **Sophie Réhault-Godbert:** Conceptualization, Methodology, Validation, Formal analysis, Investigation, Writing – original draft, Writing – review & editing, Visualization, Resources, Supervision.

## Data Availability

Recherche Data GouvSupplementary data on the effect of 2 % CO2 and 24-hour acclimatization on the quality of fertilized eggs stored for one week at 12 °C with a relative humidity of 80 % (Original data).

## References

[1] Fasenko G.M. (2007). Egg storage and the embryo. Poult. Sci..

[2] Brake J., Walsh T.J., Benton C.E., Petitte J.N., Meijerhof R., Peñalva G. (1997). Egg handling and storage. Poult. Sci..

[3] Hurnik G.I., Reinhart B.S., Hurnik J.F. (1978). Relationship between albumen quality and hatchability in fresh and stored hatching eggs. Poult. Sci..

[4] Jones D.R., Musgrove M.T. (2005). Effects of extended storage on egg quality factors. Poult. Sci..

[5] Brady K., Talbot C.C., Long J.A., Welch G., French N., Nicholson D., Bakst M.R. (2022). Transcriptome analysis of blastoderms exposed to prolonged egg storage and short periods of incubation during egg storage. BMC Genom..

[6] Pokhrel N., Genin O., Sela-Donenfeld D., Cinnamon Y. (2022). Storage temperature dictates the ability of chicken embryos to successfully resume development by regulating expression of blastulation and gastrulation genes. Front. Physiol..

[7] Guinebretière M., Puterflam J., Keïta A., Réhault-Godbert S., Thomas R., Chartrin P., Cailleau-Audouin E., Coudert E., Collin A. (2022). Storage temperature or thermal treatments during long egg Storage duration influences hatching performance and chick quality. Front. Physiol..

[8] Walsh T.J., Rizk R.E., Brake J. (1995). Effects of temperature and carbon dioxide on albumen characteristics, weight loss, and early embryonic mortality of long stored hatching Eggs1. Poult. Sci..

[9] Reijrink I.A., van Duijvendijk L.A., Meijerhof R., Kemp B., van den Brand H. (2010). Influence of air composition during egg storage on egg characteristics, embryonic development, hatchability, and chick quality. Poult. Sci..

[10] P. Blavy, INRAE Cpp-eggs. (2025), https://forge.inrae.fr/pierre.blavy/cpp-eggs/.

[11] Réhault-Godbert S., Javaloyes P. (2026). Supplementary data on the effect of 2 % CO_2_ and 24-hour acclimatization on the quality of fertilized eggs stored for one week at 12 °C with a relative humidity of 80 %. Recherche Data Gouv..

[12] R Core Team, R: A language and environment for statistical computing. R Foundation for Statistical Computing, Vienna, Austria, 2021.

[13] Bain M.M. (2005). Recent advances in the assessment of eggshell quality and their future application. World's Poult. Sci. J..

[14] Fathi M.M., Galal A., Ali U.M., Abou-Emera O.K. (2019). Physical and mechanical properties of eggshell as affected by chicken breed and flock age. Br. Poult. Sci..

[15] Percie du Sert N., Hurst V., Ahluwalia A., Alam S., Avey M.T., Baker M., Browne W.J., Clark A., Cuthill I.C., Dirnagl U., Emerson M., Garner P., Holgate S.T., Howells D.W., Karp N.A., Lazic S.E., Lidster K., MacCallum C.J., Macleod M., Pearl E.J., Petersen O.H., Rawle F., Reynolds P., Rooney K., Sena E.S., Silberberg S.D., Steckler T., Würbel H. (2020). The ARRIVE guidelines 2.0: updated guidelines for reporting animal research. PLoS Biol..

